# Cognitive appraisal of the disease and stress level in lung cancer patients. The mediating role of coping styles

**DOI:** 10.1007/s00520-022-06880-3

**Published:** 2022-02-10

**Authors:** Agata Poręba-Chabros, Magdalena Kolańska-Stronka, Piotr Mamcarz, Izabela Mamcarz

**Affiliations:** 1 Institute of Psychology, The John Paul II Catholic University of Lublin, Lublin, Poland; 2 Department of Psychology, University of Zielona Góra, Zielona Góra, Poland; 3 Department of Didactics and Medical Simulation, Medical University of Lublin, Al. Racławickie 1, 20-059 Lublin, Poland

**Keywords:** Lung cancer, Stress, Coping styles, Cognitive appraisal of disease, Health behaviour

## Abstract

**Purpose:**

The aim of the study was to provide support for the hypothesis that there was a correlation between the subjective appraisal of one’s disease and the level of stress, as well as the hypothesis that coping styles may have a mediating role on the relationship between the perception of the disease and stress level in patients diagnosed with lung cancer.

**Methods:**

The study involved 97 respondents diagnosed with lung cancer, including 50 men and 47 women. The following methods were used for the study: the Disease-Related Appraisals Scale, the Coping Inventory for Stressful Situations, and the Perceived Stress Scale. Socio-demographic data were also collected.

**Results:**

The results show that emotion-oriented coping (EOC) acts as a mediator on the relationship between the appraisal of the disease and stress level in patients diagnosed with lung cancer. A total of 4 multiple mediation models were tested.

**Conclusion:**

The research findings provide support for the hypothesis that coping style is crucial for the way patients appraise their disease and for their stress level. It is important to diagnose individual specific needs of lung cancer patients. The research results are an important source of information for those responsible for training medical staff on how to support cancer patients in their illness.

## Introduction

Nowadays, cancer diseases are one of the leading causes of death around the world, with lung cancer killing about 1.3 million people each year [1, 2]. In Poland, the number of people diagnosed with lung cancer increase yearly and of those who die as a result of it allows us to state that it is a major cause of death [3]. Lung cancer is often diagnosed when it has already reached an advanced stage, which reduces the patients’ chances of survival for more than 5 years. The high incidence of lung cancer is a consequence of growing air pollution caused by high industrialisation of large cities [4]. Lung cancer risk factors also include the following: fast pace of life, stress, smoking, passive smoking and history of cancer in the family [5]. Late diagnosis, quick course of the disease, the symptoms that are difficult to detect and the specific treatment cause that lung cancer has a highly overwhelming impact on both the physical and mental functioning of patients. Lung cancer patients experience stress [6], uncertainty and lose their sense of security [7]. It is noted that those patients who are under severe stress are more susceptible to depression and anxiety disorders [8, 9], which in turn may aggravate cancer symptoms [10]. Stress hormones may be responsible for progression of cancer, and they may accelerate the spread of cancer cells [11]. Therefore, it is important to understand the factors that influence stress levels. According to the theory of Lazarus and Folkman [12], cognitive appraisal of events as well as coping styles are factors related to stress. Research shows that coping styles mediate between perceiving a situation as stressful [13] or pre-cancer stressful life events and the perceived distress [14].

The purpose of this study was to determine whether the coping styles are the mediators of the relationship between cognitive appraisal of disease and stress level among patients with lung cancer. The article is structured as follows: first, we present theoretical issues and discuss the relationship between the cognitive appraisal of the disease, stress level and coping styles. This is followed by a description of the research procedure. Then, we present the results of the multiple mediation analysis. The article ends with a general discussion of the research results and suggestions for further research.

## Literature review and hypotheses

### From cognitive appraisal of the disease to stress

The literature states that stress occurs when individuals experience some disruption between the resources they have and their environment, and as a result, they appraise a given situation as exceeding and threatening [15]. Lung cancer is a highly delicate and specific situation that causes stress and its effects are visible in the physical, emotional, social and spiritual spheres. Lung cancer patients experience stress from the very beginning when cancer symptoms appear, throughout the process of diagnosis and then when they undergo treatment [16, 17]. In their work, researchers frequently draw a comparison between having cancer and experiencing critical life events such as death, or loss of something important [18]. In other studies, however, cancer is examined and explained in the context of traumatic stress, post-traumatic stress disorder (PTSD) and subclinical post-traumatic stress symptoms (PTSS) [19, 20]. According to the transactional theory of stress [21], the level of stress that individuals experience during lung cancer is associated with their cognitive appraisal of the disease.

In the transactional theory of stress, adaptation is defined as the ongoing cognitive and behavioural efforts to manage exceeding external and internal demands that exceed the individual’s resources and ability to adapt [12, 22, 23]. The cognitive appraisal of the disease can be considered in two ways: firstly, as the patient’s knowledge about their disease, and secondly, as the subjective appraisal of how it will influence their life. In the primary assessment, according to Lazarus and Folkman’s theory [21], a situation can be classified into three categories; namely, it can be considered to be a (1) threat, (2) harm or a (3) challenge. In the first category, illness is understood as an obstacle to satisfying needs and desires, something that hinders our activity. The category of harm is related to feelings of injustice and unfairness because of getting ill. In the third category, a disease is treated as one of many difficult life events that must be dealt with [24]. Various authors have pointed out that there may be more than just three categories of cognitive appraisals related to one’s disease (DRAs) [25, 26]. It is emphasised that going through illness may elicit many other associations [27]; therefore, complementary categories are distinguished; i.e., illness can be viewed as an (4) obstacle/loss, because it places many limitations on the patient; (5) profit—obtaining some secondary gain from being ill; e.g., interest, care; (6) value—illness may have a deeper meaning for the patient, revealing some values or realities that they have not paid attention to before; and (7) significance—to what extent illness is an important life event for the patient. These categories are not mutually exclusive; they may exist side by side, even though they may seem contradictory, which is probably a result of the complex and dynamic nature of disease and stressor.

Research by Bigatti, Steiner and Miller [28] showed relationships between harm/loss appraisal, coping strategies and depressive symptoms in women with breast cancer. We also know that in a situation exceeding the adaptive capacity of an individual, people use defense mechanisms involving the avoidance or reduction of threatening emotions, mainly anxiety and fear. Defense mechanisms help sustain mental stability but if used excessively or inadequately, they may cause problems at any stage of oncological diseases, such as distorted perception of the situation [29]. In the case of denial, distressing information (e.g. unfavourable prognosis, poor test results) is ignored and replaced with a more harmless interpretation that is often not relevant to the patient’s current situation. In the case of repression, psychological distress is initially realised and then forgotten. However, repressed feelings can unconsciously influence a person’s behaviour patterns [30]. Recent scientific findings suggest that defense mechanisms have an impact on cancer patients’ physical and psychological conditions, as well as their attitudes toward treatment options [31]. Research indicates that illness awareness is problematic for patients diagnosed with lung cancer [16, 17], and therefore, despite low scores in the cognitive appraisal of the disease, they may experience high levels of stress and other negative consequences. However, the very fact of having cancer is connected with the occurrence of stress [32]. Thus, we formulate a hypothesis:

- H1: Cognitive appraisal of the disease is negatively correlated to stress level.

### From cognitive appraisal of the disease to stress coping styles

Cognitive appraisals and coping styles are two key psychological processes that influence the consequences of a given stressful situation [32]. Coping styles involve diverse efforts made by people to solve a stressful situation (i.e. problem-oriented styles) or to alleviate negative emotions experienced as a result of a stressful situation (i.e. emotion-oriented styles). When a stressor occurs, people may generate different cognitive appraisals and undertake diverse coping strategies, some of which may be highly adaptive and some of which may be maladaptive. Adaptive cognitive appraisals are those that help the person reduce the negative consequences of stress, whereas maladaptive cognitive appraisals and coping strategies are those that fail to protect the person from the negative consequences of stress [33, 34]. Research results indicate that there is a strong correlation between the cognitive appraisal of illness and the process of adapting to it, on the basis of which it is possible to determine stress level. DRAs can directly influence the strategies a patient chooses to cope with disease-related stress. The effectiveness of coping, in turn, translates into the achieved levels of adaptation to living with the disease [35]. Adaptation to cancer is elicited by emotions that differ depending on the subjective meaning attributed to the disease [36]. It has been noted that there is a positive correlation between avoidant and emotion-oriented coping and the helplessness-hopelessness approach and anxiety preoccupation [37]. It can be concluded that people with higher level of cognitive appraisal of their disease will be more likely to approach the situation as a task even if this appraisal is difficult for them. On the other hand, lower cognitive appraisal is associated with a more emotion-oriented processing of information [38]. We expect that:

- H2: Cognitive appraisal of the disease is correlated to stress coping styles: (H2.1.) The higher the score on the Disease-Related Appraisals Scale, the higher the score on task-oriented style, and (H2.2.) avoidant style, while (H2.3.) the lower the score on the Disease-Related Appraisals Scale, the higher the level of engaging in emotion-oriented coping.

### From coping styles to stress

Coping styles are related to the process in which stress occurs and to many other factors which modify the stress relationship and which are often considered as variables [36, 39]. Research has shown that the styles related to the fighting spirit, acceptance and positive re-evaluation were the most adaptive for recovering among cancer patients [40]. Based on the transactional theory of stress, other researchers identify three styles of coping with stress: task-oriented, emotion-oriented and avoidant style. Lazarus and Folkman emphasise that the coping style should be understood as a collective pattern characterised by moderate constancy and consistency in a given person [12]. The concept of coping style does not emphasise total constancy or rigidity in the process of coping with stress. Therefore, in the context of cancer, the coping style should be understood as some kind of disposition, which is flexible and allows the individual to change strategies and adjust them to specific conditions [41].

The most recent studies among patients diagnosed with lung cancer indicate that those patients most frequently activate adaptive methods of coping with stress caused by their disease [42]. The task-oriented style is observed predominantly, while avoidant or emotion-oriented styles are less common. It is pointed out that patients who employ task-oriented strategies are less likely to feel helpless. Other studies have found that the type of coping style used by those who have been cured of cancer may be a predictor of their quality of life [43]. In studies of people diagnosed with lung cancer, the task-oriented coping style may reduce depressive symptoms, whereas the avoidant style can predict more severe depressive symptoms [17]. Positive re-framing increases the likelihood that cancer patients will be able to identify advantages of their experience, such as post-traumatic growth and finding benefit [44, 45]. For example, as a result of being diagnosed with cancer, they may discover greater spiritual significance or deepen interpersonal relationships. Identifying those benefits is in turn correlated with lower perceived cancer-related stress [44, 45]. Moreover, previous research shows that the intensity of stress symptoms may be predicted on the basis of appraisal of the disease in terms of stress and on the basis of self-assessment of coping with cancer [46]. Based on previous research, we expect that:

- H3: Coping styles are related to stress level: (H3.1.) Stress level decreases with a higher score in task-oriented style, whereas it increases with a higher score in emotion-oriented coping (H3.2.) and in avoidant coping (H3.3.).
- H4: Coping styles mediate the relationship between cognitive appraisal of the disease and stress.

## Materials and methods

### Respondents and procedure

Before the research was started, the University Ethical Board in accordance with the ethical standards as laid down in the 1964 Declaration of Helsinki and its later amendments or comparable ethical standards approved the methods and procedures (no. 02/06/16). The research was conducted at the Independent Public Clinical Hospital No. 4 in Lublin (after the consent of the hospital administration to conduct the study, based on the opinion of the head of the oncology ward and the ethics team), and involved cancer patients diagnosed with lung cancer. The results obtained from 97 respondents, including 50 men (51.5%) and 47 women (48.5%), were examined. The respondents’ age ranged from 35 to 84 (SD: 7.822, min: 35, max: 84), with the average age being 64.84. The situation of respondents varied in terms of time since diagnosis, severity of diagnosis and stage of treatment. The questionnaire was administered individually in direct contact with patients. Patients were asked whether they would like to participate in the survey voluntarily after that they were given the paper consent form and questionnaires. They were informed about the purpose of the research and that the research was anonymous. The consent form included also the contact to the leading investigator and information that filling in the questionnaire was equivalent to consenting to participate in the study. The interviewer did not know the respondents. A face-to-face meeting with a patient during which the questionnaire data was collected lasted between 30 and 60 min. The characteristics of the sample are included in Table 1.

**Table 1:** Characteristics of the research group

Variable		*N*	%
Sex	Male	50	51.5
	Female	47	48.5
	Total	97	100.0
Smoking tobacco	Yes	48	49.5
	No	49	50.5
	Total	97	100
Type of cancer	Non-small cell cancer	81	83.5
	Small cell cancer	16	16.5
Marital status	Single	4	4.1
	Married	68	70.1
	Widower/widow	19	19.6
	Divorced	6	6.2
	Total	97	100.0
Place of residence	Village	38	39.2
	Small town	18	18.6
	Medium-sized city	14	14.4
	Big city	28	26.8
	Total	96	99.0
Professional status	Active	18	18.6
	On sick leave	5	5.2
	Retirement pension	61	62.9
	Disability pension	11	11.3
	Total	95	97.9
Treatment used	Chemotherapy	93	95.9
	Radiotherapy	16	16.5
	Surgical treatment	9	9.3
Number of chemotherapy treatments	1–3	36	39.6
	4–7	46	47.4
	8 and more	9	9.3
	Total	91	93.8
Time since diagnosis	0–54 months	23	27.8
	55–68 months	52	53.6
	69 and more months	9	9.3

### Measurement of variables

#### Appraisal of one’s disease

The Disease-Related Appraisals Scale (DRAS) developed by Steuden and Janowski was used to measure the subjective perception of the disease. It is a questionnaire tool that tests the subjective meanings attributed by patients to their disease. It consists of 47 statements, to which the respondents mark their answers on a 5-point scale: where 5 yes, 4 rather yes, 3 hard to say, 2 rather no and 1 no. The scale consists of 7 subscales: disease appraisal as (1) threat, (2) benefit, (3) loss, (4) challenge, (5) harm, (6) value and (7) significance. Statistical analyses showed that the internal reliability (α) on particular scales ranged between 0.64 and 0.87. Correlations between scales are independent [35].

#### Styles of coping with stress

The Coping Inventory for Stressful Situations (CISS) developed by Endler and Parker (1990) was used to measure the coping styles variable. It consists of 48 statements that represent different behaviours activated in stressful situations. The questionnaire consists of three subscales corresponding to three coping styles: SSZ (TOS) task-oriented style; SSE (EOS) emotion-oriented style; and SSU (AS) avoidant style. Each of these sub-scales consists of 16 items and the respondents can score from 16 to 80 points in each. The original questionnaire had high internal consistency reliability of particular scales (coefficients ranging between 0.78 and 0.90), and satisfactory test re-test reliability (correlation coefficients between two questionnaires conducted at an interval of 2 or 3 weeks ranged from 0.73 to 0.80). Cronbach’s reliability coefficients α are high and range from 0.84 for the emotion-oriented style subscale to 0.90 for the task-oriented style subscale.

#### Stress level

The Perceived Stress Scale (PSS-10) developed by Cohen et al. was used to measure the stress level variable [47]. The scale was designed to assess an individual’s response to a stressful situation in which they find themselves. The total scale score is the sum of all items, and can take values from 0 to 40, with higher scores indicating higher perceived stress. In the case of the stress scale, the results in this study were obtained by calculating the total value obtained for all questions. Cronbach’s internal reliability coefficient α is high and stands at 0.91.

### Statistical analysis method

**Fig. 1:**
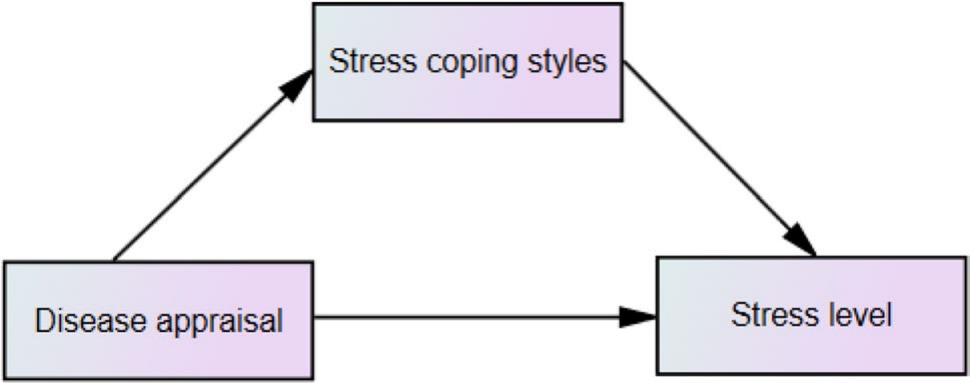
Coping styles as the mediator on the relationship between the cognitive appraisal of the disease and stress level

A multiple mediation model (Fig. 1) was tested by using Hayes (2013) PROCESS macro (model 4) with the total scores on the items for appraisal of one’s disease dimensions, Styles of coping with stress factors and stress level. The analysis assessed (1) the effects of appraisal of one’s disease dimensions on stress level, (2) the effect of appraisal of one’s disease dimensions on styles of coping with stress dimensions and (3) the effect of Styles of coping with stress dimensions on stress level. The 95% bias-corrected confidence interval from 5000 resamples was generated by the bias-corrected bootstrapping method to evaluate the statistical significance of the correlation and effects.

## Results

### Descriptive statistics

Data analysis revealed that stress level was statistically significantly correlated with disease appraisal, in every aspect except benefit (95% CI = [− 0.36, 0.03]), challenge (95% CI = [− 0.35, 0.04]) and value (95% CI = [− 0.31, 0.09]). As expected, a positive correlation was found between the task-oriented style and disease appraisal: threat (95% CI = [0.10, 0.46]), benefit (95% CI = [0.13, 0.49]), loss (95% CI = [0.23, 0.56], harm (95% CI = [0.27, 0.59] and significance (95% CI = [0.16, 0.52]. Negative associations were also obtained between emotion-oriented style and disease appraisal: threat (95% CI = [− 0.69, − 0.41]), benefit (95% CI = [− 0.50, − 0.14]), loss (95% CI = [− 0.58, − 0.25], challenge (95% CI = [− 0.48, − 0.12]), harm (95% CI = [− 0.66, − 0.37], value (95% CI = [− 0.39, − 0.00]) and significance (95% CI = [− 0.55, − 0.21]. Only disease appraisal as significance correlates with avoidant style (95% CI = [0.06, 0.44]) (Table 2).

**Table 2:** Means, standard deviations and Pearson’s r correlations between variables for the whole sample

Variable	M	SD	1	2	3	4	5	6	7	8	9	10
1. DA: Threat	14.76	7.33										
2. DA: Benefit	23.31	4.75	0.51**									
3. DA: Loss	21.56	6.75	0.69**	0.55**								
4. DA: Challenge	12.74	3.64	0.39**	0.50**	0.43**							
5. DA: Harm	24.33	6.14	0.54**	0.39**	0.65**	0.32**						
6. DA: Value	20.63	7.00	0.28**	0.35**	0.19	0.41**	0.06					
7. DA: Significance	8.62	3.58	0.66**	0.24*	0.45**	0.33**	0.40**	0.20				
8.Stress level	57.30	8.18	− 0.56**	− 0.17	− 0.45**	− 0.16	− 0.48**	− 0.11	− 0.32**			
9. TOS	38.66	8.91	0.29**	0.33**	0.41**	0.17	0.44**	− 0.05	0.35**	− 0.32**		
10. EOS	44.89	6.00	− 0.57**	− 0.34**	− 0.43**	− 0.31**	− 0.53**	− 0.20*	− 0.39**	0.71**	− 0.44**	
11. AS	17.33	6.60	0.12	− 0.08	0.12	− 0.13	0.13	− 0.12	0.26*	− 0.07	0.05	0.02

*DA* disease appraisal, *TOS* task-oriented style, *EOS* emotion-oriented style, *AS* avoidant style.

*p < 0.05.

**p < 0.01.

***p < 0.001.

### Mediation

The next step involved multiple mediation analysis, in which we tested the mediation effect using the bootstrapping method. The mediating effect was examined only for significant relationships between aspects of cognitive appraisal of one’s disease and stress level. We examined how coping styles mediate the relationship between the disease appraisal (threat, loss, harm, significance) and the patient’s stress level.

**Fig. 2:**
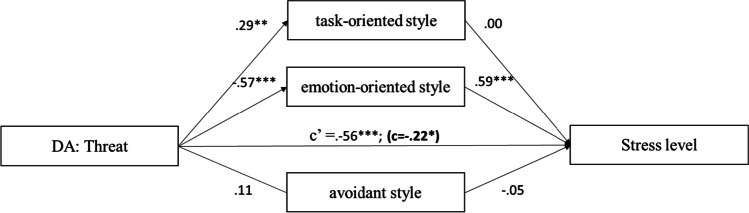
The mediation model of the relationship between threat and stress level. *Note.* The figure presents the standardised coefficients; c′ direct effect *X* to *Y*; c direct effect *X* to *Y* with mediator in model; *p < 0.05; **p < 0.01; ***p < 0.001

The results (Fig. 2) showed that disease appraisal as threat positively predicted task-oriented style (95% CI = [0.10, 0.54]) and negatively for emotion-oriented style (95% CI = [− 0.90; − 0.49]). The obtained models indicate that emotion-oriented style is a partial mediator of the relationship between threat and stress level.

**Fig. 3:**
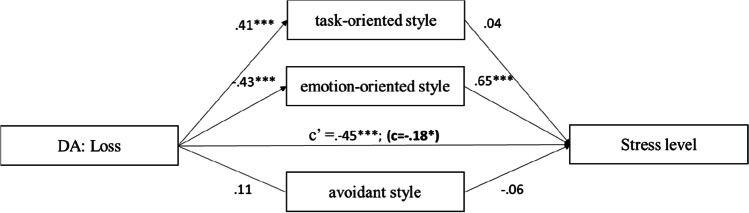
The mediation model of the relationship between loss and stress level. *Note.* The figure presents the standardised coefficients; c′ direct effect *X* to *Y*; c direct effect *X* to *Y* with mediator in model; *p < 0.05; **p < 0.01; ***p < 0.001

The results (Fig. 3) showed that disease appraisal as loss positively predicted task-oriented style (95% CI = [0.27, 0.72]) and negatively for emotion-oriented style (95% CI = [− 0.81; − 0.32]). The obtained models indicate that emotion-oriented style is a partial mediator of the relationship between loss and stress level.

**Fig. 4:**
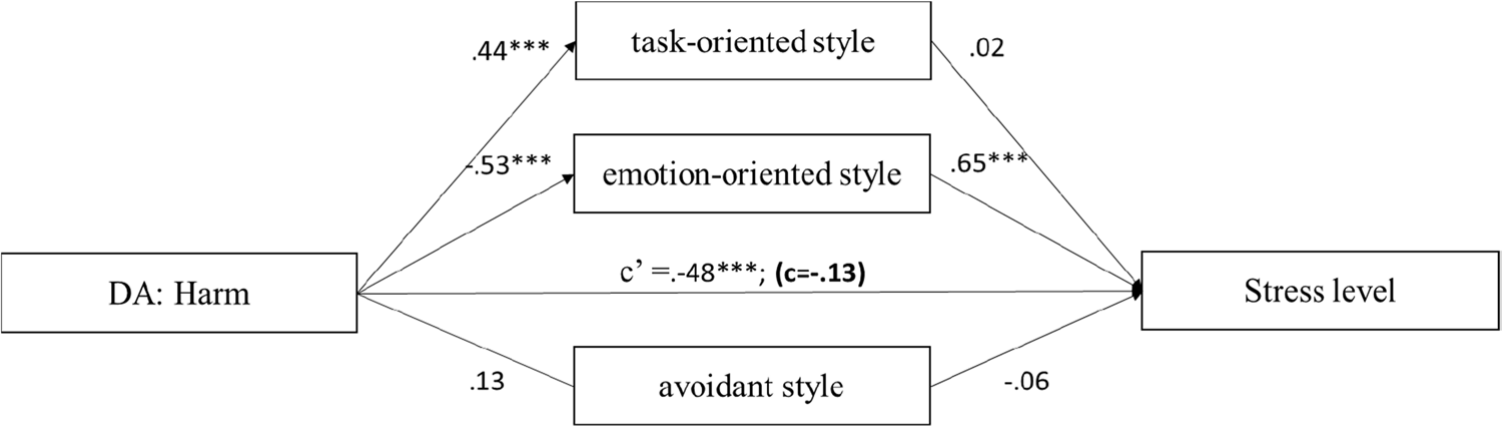
The mediation model of the relationship between harm and stress level. *Note.* The figure presents the standardised coefficients; c′ direct effect *X* to *Y*; c direct effect *X* to *Y* with mediator in model; *p < 0.05; **p < 0.01; ***p < 0.001

The results (Fig. 4) showed that disease appraisal as harm positively predicted task-oriented style (95% CI = [0.35, 0.83]), and negatively for emotion-oriented style (95% CI = [− 1.02; − 0.52]). The obtained models indicate that emotion-oriented style is a full mediator of the relationship between harm and stress level.

**Fig. 5:**
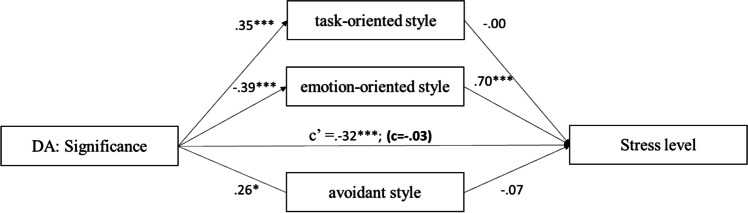
The mediation model of the relationship between significance and stress level. *Note.* The figure presents the standardised coefficients; c′ direct effect *X* to *Y*; c direct effect *X* to *Y* with mediator in model; *p < 0.05; **p < 0.01; ***p < 0.001

The results (Fig. 5) showed that disease appraisal as significance positively predicted task-oriented style (95% CI = [0.37, 1.24]), avoidant style (95% CI = [0.10, 0.76]) and negatively for emotion-oriented style (95% CI = [− 1.44; − 0.50]). The obtained models indicate that emotion-oriented style is a full mediator of the relationship between harm and stress level.

No mediating effect of task-oriented style and avoidant style was observed for the relationship between the disease appraisal and the patient’s stress level.

## Discussion

The purpose of this study was to examine the relationship between the appraisal of the disease and stress level and to determine the potential mechanism of how the appraisal of the disease could predict stress from the perspective of coping styles. As expected, the results showed a negatively correlation between appraisal of the disease and stress level, which supports hypothesis 1. Becoming aware of their disease is difficult for patients diagnosed with lung cancer [16, 17]. The disease carries many different connotations [39] and is sometimes compared to post-traumatic stress [19, 20]. The study showed that lung cancer patients who perceived the disease at a low level as threat, loss, harm or significance experienced high levels of stress. It may be related to defense mechanisms (denial/repression) that affect the cognitive appraisal of the disease and can cause negative somatic and psychological consequences [30].

In our research, we also attempted to demonstrate the relationship between the appraisal of the disease and coping styles (H2). The cognitive appraisal of the disease as threat, benefit, loss, harm and significance turned out to be positively related to task-oriented style. These results indicate that the more aware patients are of their disease, even if they have very negative associations with it, the more likely they are to engage in task-oriented coping, which may turn out to be very adaptive [34]. On the other hand, the lower the level of cognitive appraisal of the disease in each category, the more likely patients are to engage in emotion-oriented coping [33]. In the case of avoidant style, there was only a correlation with the appraisal of the disease as significance.

The research also answers the question concerning the relationship between coping style and stress level in lung cancer patients. We have observed a positive association between emotion-oriented style and stress level, and a negative association between task-oriented style and stress level. No association has been noted between avoidant style and stress. These results are consistent with the literature to date; patients who use task-oriented strategies are less likely to feel helpless [42], and show a lower level of depressive symptoms [43]. On the other hand, the use of emotion-oriented style is associated with the helplessness-hopelessness approach and anxiety preoccupation [42].

It was shown that (1) the emotion-oriented style was a partial mediator in the relationships between the appraisal of the disease as a threat, loss, harm and a full mediator in the relationship between the cognitive appraisal of disease as significance and stress level; and (2) the task-oriented style and avoidant style (AS) did not play a mediating role in the relationship under study, which partially supports hypothesis 4.

These findings indicate that emotion-oriented style plays a different role in the perceived level of stress in lung cancer patients. An important correlation was noted in each of the indicated cases of mediation: when a respondent engaged in emotion-oriented coping, the correlation between the appraisal of the disease and stress level decreased. Therefore, to understand the stress mechanism of lung cancer patients, it is important to take into account not so much their cognitive appraisal of the disease, but above all emotion-oriented coping. A higher cognitive appraisal of the disease as threat, loss, harm and significance is associated with a decrease in emotion-oriented coping, which in turn results in a lower level of stress. This can be explained in the following way: since patients consciously appraise their disease as some negative state, they are able to activate the resources needed to cope with it and to make efforts to improve the way they function using adaptation strategies, which leads to reducing stress (by taking a partial control over disease). On the other hand, patients whose cognitive appraisal of their disease may be distorted, which makes them deny that they are ill, are more likely to engage in emotion-oriented coping (requiring more resources, but not very adaptive), which results in an increased level of stress. It is possible that in this way, they distance themselves from the cognitive appraisal of their disease to protect a sense of stability. Although the symptoms, limitations and difficulties they have to face are undeniable, they cannot deal with them adaptively, because “the disease does not matter that much”, so it is not possible to control what is happening. As a result, their level of stress increases.

Our research findings make several important theoretical contributions to the understanding of cancer-related stress. Firstly, they support the results of previous empirical studies that showed a strong correlation between the patients’ appraisal of their disease and their stress level [36]. The obtained results are also in line with studies conducted among patients suffering from multiple sclerosis, which concerned the mediating function of coping strategies between the cognitive appraisal of the disease and post-traumatic growth [48]. Researchers showed that both the strategy of anxiety preoccupation and that of helplessness/hopelessness acted as mediators [49]. Other studies that involved cancer patients as a research group also showed that emotional regulation acted as a mediator [50], which concurs our findings.

## Limitations and suggestions for future research

Our research supports the hypotheses that we put forward; however, it has some limitations, which are worth pointing out. We adopted a cross-sectional study method. Yet, it seems that longitudinal studies are desirable to get a thorough understanding of the mediating role of coping styles. In the case of lung cancer, it is important to determine the time of treatment or the stage of the disease, as this may greatly influence coping styles, cognitive appraisal and stress levels. Taking into account the nature of lung cancer and high mortality rates among cancer patients, this may be an extremely difficult task, but not impossible. Another limitation of our re-search was that the study group was confined to patients in just one hospital ward, and in our analyses, we did not take into account the specificity of its functioning. Determining the specifics of this particular ward could provide some valuable information regarding, for example, the quality of doctor-patient relationship or support provided to patients. This could also contribute to a better understanding of the phenomenon under study and to determining whether it is repeatable or not. Future research could also consider how the appraisal and coping style change by examining them in longitudinal studies.

## Conclusions

Since we are aware of a link between stress and the course of cancer [11], it is crucial to identify those factors that can reduce stress. The research results demonstrate the significant role of coping styles in the perception of illness and the level of stress. Appraising illness appropriately and activating the right coping style may significantly contribute to lowering the patient’s stress levels. Therefore, it is important that lung cancer patients are provided with regular psychological support in addition to cancer treatment they receive.

Numerous interventions aimed at reinforcing action-oriented coping strategies have been developed for both cancer and non-cancer populations [51]. Specific psychotherapeutic approaches (e.g. cognitive behavioural therapy, or cognitive behavioural stress management) are used with cancer patients to engage them in an adaptive coping (e.g. by using behavioural activation; identifying and combating cognitive distortions; and dealing with harmful, negative core beliefs about themselves and the world around). These could help reduce stress levels in cancer patients.

## Data Availability

The data is available in “figshare” repository: https://doi.org/10.6084/m9.figshare.13681828.v1.
